# Association of Hepatitis C Virus Infection with Type II Diabetes in Ethiopia: A Hospital-Based Case-Control Study

**DOI:** 10.1155/2012/354656

**Published:** 2012-09-23

**Authors:** Solomon Ali, Solomon Abera, Adane Mihret, Tamrat Abebe

**Affiliations:** ^1^Department of Medical Laboratory Sciences and Pathology, College of Public Health and Medical Sciences, Jimma University, Jimma, Ethiopia; ^2^Department of Microbiology, Immunology and Parasitology, College of Health Sciences, Addis Ababa University, Addis Ababa, Ethiopia; ^3^Department of Microbial, Cellular and Molecular Biology, College of Natural Sciences, Addis Ababa University, Addis Ababa, Ethiopia; ^4^Armauer Hansen Research Institute, Addis Ababa, Ethiopia

## Abstract

*Background.* Chronic hepatitis C virus (HCV) has become the global “epidemic” with an estimated 123 million people currently infected worldwide. As the same time diabetes is also rapidly emerging as a global health care problem that threatens to reach pandemic levels by 2030. *Objective.* To investigate the magnitude of HCV infection in type II diabetes as compared to controls. *Methodology.* A case control study design was conducted at Jimma University Specialized Hospital from May to June 2010. A total of 604 study subjects were included in this study. Sociodemographic and risk factor data were collected by questionnaire. From serum sample, HCVAb screening was done by rapid antibody screening test. Liver functioning tests and total cholesterol tests were done by Dr. Lange LP 800 spectrophotometer. *Results.* The prevalence of HCV in type II diabetes and nondiabetic controls was 9.9% and 3.3%, respectively. In multivariate analysis, HCV seropositives have high risk of developing diabetes as compared with seronegatives (AOR = 2.997, 95% CI: (1.08, 8.315)). *Conclusion.* In this study, we found a positive association between past HCV infection and type II diabetes. As we did not perform HCV RNA test, we could not assess the association with HCV viremia.

## 1. Introduction

Hepatitis C virus (HCV), RNA single strand positive sense genome virus, was first recognized as a separate disease entity in 1975 when the majority of transfusion-related hepatitis were found not to be caused by the only two hepatitis viruses recognized at that time that is Hepatitis A virus and Hepatitis B virus. The disease at that time was called “non-A non-B hepatitis. The discovery of hepatitis C genome in 1989 has now led to the realization that this virus is a major health problem worldwide [1–3]. 

HCV is most efficiently transmitted through transfusion of infected blood, transplantation of infected organs, and sharing injection drug equipments [4]. The majority of persons with newly HCV infection are asymptomatic [2, 4]. Only 20% of them develop symptoms such as fatigue, abdominal pain, poor appetite, or jaundice, usually within 4–12 weeks. Apart from these over 50% of those infected individuals will suffer from chronic hepatitis, which may ultimately lead to severe liver disease, cirrhosis, or even the development of hepato-cellular carcinoma and death [5, 6].

WHO estimated the prevalence of HCV infection to be 2%, representing 123 million people [7]. HCV is the leading cause of liver transplantation in developed countries, and the most common chronic blood borne infection in the USA [8]. According to a press released by WHO in April 1998; prevalence of HCV was within the range of 0.5–10% in population samples around the world [1]. It is only recently with the advent of modern testing methods in the 1990's, that the epidemiology of HCV could be studied better. As a result, high rates of HCV infection in many areas of the world are just being recognized as a significant public health problem. From these high rate areas sub-Saharan Africa has the highest WHO estimated regional HCV prevalence (5.3%) [9].

In Ethiopia, there are few studies done in relation to prevalence of HCV infection. Recent study done in Gondar among blood donors reported 0.7% HCV prevalence [10]. Previous studies estimated the prevalence of HCV to be 1.4% [11, 12]. As per WHO, the prevalence of HCV in Ethiopia is estimated to be within a range of 2–2.9% [7].

Infection with HCV affects not only the liver but the extra hepatic tissues as well. It may combine with many unrelated diseases and morbid conditions. A number of extra hepatic manifestations have been recognized such as disorders involving renal, dermatologic, hematologic, rheumatologic systems, and endocrine abnormality like diabetes mellitus [1, 11]. 

On those days, the number of people with diabetes is increasing due to population growth, aging, urbanization, increasing prevalence of obesity, and physical inactivity [12]. Diabetes is rapidly emerging as a global health care problem that threatens to reach pandemic levels by 2030. The number of people with diabetes worldwide is projected to increase from 171 million in 2000 to 366 million by 2030. This increase will be most noticeable in developing countries, where the number of people with diabetes is expected to increase from 84 million to 228 million [12]. According to the WHO; Southeast Asia and the Western Pacific regions are at the forefront of the current diabetes epidemic, with India and China facing the greatest challenges. In these countries, the incidence and prevalence of type II diabetes among children are also increasing at an alarming rate, with potentially devastating consequences [13, 14].

The etiology of type II diabetes is still not completely understood. However, on top of genetic, biologic, and demographic factors, recent studies have also suggested as HCV infection could also be associated with type II diabetes. Clinical and experimental data suggested a direct role of HCV in the perturbation of glucose metabolism. HCV may alter glucose homeostasis by its direct action, or via indirect mechanisms such as through cytokine stimulation. Apart from this, HCV infection induces cytotoxic T cell response which damages hepatocytes. This liver perturbation and damage elevates liver cell damage marker enzymes like Aspartate transaminase (AST) and Alanine transaminase (ALT) [15, 16].

The association between diabetes mellitus and HCV infection has only recently been posed in the international literature and remains unexplained. Several investigators have approached the relation between type II diabetes and HCV either by measuring the prevalence of HCV markers among populations of diabetic patients or by measuring glucose intolerance among HCV infected populations [13, 17].

Currently the association between hepatitis C virus (HCV) infection and type II diabetes had been reported in a number of clinical studies though conflicting results are reported [18, 19].

To the best of our knowledge, there is no documented report about the prevalence of HCV among *n* diabetic patients in Ethiopia. But there is one cross-sectional study done in Tikur Anbessa Hospital, GI clinic to determine glucose intolerance on HCV infected subjects. This research indicated that the prevalence of type II diabetes is to be 34% in HCV infected patients. Thus, this study is designed to determine and evaluate the prevalence of anti-HCV antibody among diabetic patients visiting Jimma University Specialized Hospital (JUSH), as compared to nondiabetic controls.

## 2. Materials and Methods

A case control study was conducted in JUSH, Jimma, Ethiopia. JUSH has 11 wards, about 426 beds and 319 health professionals. It is teaching hospital providing services to over 1 million patients. The diabetic clinic of this hospital is currently giving follow up treatment service for 2000 registered diabetic patients. In this study cases and controls were defined as follows.

**Cases**

Known, diagnosed diabetic patient who is having followup at JUSH diabetic clinic, and newly diagnosed diabetic patients who were referred to the diabetic clinic during the data collection period.

**Controls**

Nondiabetic, voluntary counseling, and testing (VCT) clients of JUSH with FBS < 100 mg/dl or RBS <126 mg/dL, nonhypertensive, and with no overt liver disease.

The study protocol was ethically cleared by Addis Ababa University, Faculty of Medicine Institutional Review Board. Convenient and random sampling techniques were employed in this study, that is, 304 consecutive diabetic patients and randomly selected 300 VCT clients were included by considering diabetic patients as a homogenous group. After explaining the purpose of the study, consent was obtained from each study participant prior to data collection. Hired physician examined the controls for hypertension, overt liver disease and sent legible controls to the laboratory for screening to RBS/FBS test which was done by senior laboratory technologist. Two senior nurses (one from diabetic clinic and other one from VCT clinic) collected the sociodemographic data and other risk factor data from volunteer and legible participants by using predesigned, pretested and structured questionnaire. Those participants were sent to the laboratory for investigation.

In the laboratory, Senior Laboratory Technologist has collected 5 mL of venous blood sample by sterile disposable vacutainer tube system and left for 30 minute to facilitate clotting and then the clotted blood was centrifuged to separate the serum from blood. The serum was divided in to two aliquots. One of the aliquot was used for Anti-HCV antibody screening as per manufacturer's instruction (Anti-HCV cassette, Linear Chemicals SL, Barcelona, Spain). The other aliquot was used for LFT and cholesterol tests by using Dr. Lange LP 800, Germany spectrophotometer. 

The result was noted on laboratory data collection format sheet. From these precoded checked data was entered to SPSS windows 17 and analysis was done as per the type of data; for categorical data, descriptive statistics, univariate, multivariate logistic regression analysis, *P* value, and odds ratio (OR) computation was done. For quantitative data unpaired *t*-test was employed to compute the mean values of AST, ALT, and cholesterol from cases and controls. *P* value <0.05 were taken as statistically significant.

## 3. Results

In this study, a total of 604 participants (304 subjects with diabetics and 300 nondiabetic controls) were included. Of the diabetic subjects, 188 (61.8%) were males and the rest 116 (38.2%) were females. On the other hand, 170 (56.7%) males and 130 (43.3%) females were included from nondiabetic controls. The male to female ratio of diabetics and non diabetic controls was 1.62 and 1.3, respectively. There is no significant difference in sex distribution among diabetic patients and their non diabetic controls. If we see study participant's nature of the work; 54.3% of diabetics and 75% of non diabetic controls had nonsedentary nature of work. This non sedentary nature of work is protective for diabetes (AOR = 0.261, 95% CI: (0.150, 0.451)) (Table 1).

Concerning BMI, majority of diabetics (56.6%) and non diabetics (87%) were within a normal range of 18.5–24.9 kg/m^2^ value. In multivariate analysis, it was indicated that subjects with BMI of 25–29.9 Kg/m^2^ had three times chance to become diabetic as compared with <18.5 BMI (AOR 3.137, 95% CI: (1.059, 9.286)) (Table 1).

The prevalence of HCV in diabetes and non diabetic controls was found out to be 9.9% and 3.3%, respectively (*P* = 0.002). There was statistically significant difference in distribution of HCV among diabetic and non diabetic controls. In multivariate analysis of binary logistic regression, it was indicated that HCV seropositive subjects had almost three times risk of developing diabetes as compared to HCV seronegatives after adjusted to sex, age, nature of the work, and BMI (AOR = 2.997, 95% CI: (1.08, 8.315)) (Table 1).

With regard to HCV risk factors which includes ear piercing, body piercing, tattoo, tooth extraction, hospital admission, history of transfusion and contact with jaundiced person; none of these risk factors were significantly associated with HCV seropositivity in both diabetic and non diabetic controls (*P* > 0.05) (Table 2).

The unpaired *t*-test result indicated that there was statistical significant difference in age between diabetic and non diabetic controls (17.456 years) (*P* = 0.000 95% CI: (15.49, 19.42)). The mean height in meter of diabetic and non diabetic controls was 1.68 ± 0.52 and 1.64 ± 0.092, respectively. There is no statistically significant difference in height between diabetics and non diabetic controls (*P* = 0.199, 95% CI: (0.02, 0.1)). The mean weight in kg of diabetic and non diabetic controls was 62.2 ± 13.5 and 59.5 ± 7.4, respectively. There is significant difference in weight between diabetic and non diabetic controls with mean difference of 2.62 kg (*P* = 0.003, 95% CI: 0.88, 4.38)) (Table 3).

In relation to BMI, there is statistically significant difference between diabetic and non diabetic subjects with mean difference of 0.69 kg/m^2^ (*P* = 0.022, 95%, CI: (0.098,1.28)). The LFT mean difference of AST between the diabetic and non diabetic controls was 1.56 U/L which is statistically significant (*P* = 0.037, 95% CI: (0.092, 3.03)). However, the mean ALT difference between diabetic and non diabetic controls was 0.52 U/L which was not statistically significant (*P* = 0.656, 95% CI: (−1.783, 2.83)). The mean cholesterol concentration difference between diabetic and non diabetic controls was 65.33 mg/dl which was statistically significant (*P* = 0.000, 95% CI: (49.33, 81.34)) (Table 3).

## 4. Discussion

In this study, we used HCV cassette antibody test (Linear Chemicals SL, Barcelona, Spain) to detect anti- HCV antibody from serum of the study participants. As a limitation; we could not perform HCV RNA or protein detection which is almost impossible to test here due to the very high cost and facility. 

The seroprevalence rate of anti-HCV in type II diabetic patients and non diabetic controls was found to be 9.9% and 3.3%, respectively. In comparison, individuals with type II diabetes showed a 3.17 times higher rate of HCV seropositivity by univariate analysis. Even after adjustment for other risk factors for type II diabetes such as age, sex, education level, sedentary life style, and BMI by multiple logistic regression, individuals with type II diabetic group still showed 2.997 time higher rate of HCV sero positivity as compared to non diabetic controls (AOR 2.997, 95% CI: (1.08, 8.315)). 

The finding of this study exploring high rate of HCV seropositivity in diabetic patients is in agreement with other studies reported before. Reports from Africa, North America, Europe, and the Middle East consistently demonstrated an increased prevalence of HCV among diabetic patients compared with individuals without diabetes. Uncontrolled studies done in Nigeria, James et al., 2009, Pakistan, Sobia et al., 2007, Japan, Michiaki et al., 2003, Pakistan, Qureshi et al., 2002, Italy, Sangiorgio et al., 2000 and United kingdom, Gray et al., 1995 with the objective of determining the prevalence of HCV in different diabetic groups revealed higher prevalence of HCV that range from 5.1–36% with *P* < 0.05. In addition various controlled studies done in Taiwan by Hua-Fen et al., 2006, Taiwan by Chong-Shan et al., 2003, USA by Mehta et al., 2000, and New York by Andrea et al., 2003 to determine the association between HCV and diabetes revealed significant association between HCV and diabetes with odds ratio (OR) of 2.87, 3.3, 3.77, and 2.9, respectively at 95% CI. These findings are also in agreement with our finding [1, 4, 13, 20–26]. 

It is not possible to forward easily for the cause of this increased prevalence of HCV in diabetic patients. But there are two possibilities; the first possibility might be in association with increased vulnerability of diabetic patients for HCV as a result of repeated exposure for finger prick injury, daily insulin injection and immune compromised state as result of diabetes. The other possibility might be due to the direct and/or indirect effect of HCV infection on glucose metabolism. In these respects, different literatures suggest the mechanisms of development of glucose intolerance in HCV infected patients though it is not well understood.

As per experimental researches, it seems that the virus itself through its core protein can modify the metabolic profile of HCV infected patients which leads to development of type II diabetes mellitus. Mechanistic studies have revealed that HCV encoded proteins may cause postreceptor defects in insulin receptor substrate 1 (IRS-1). It may also associate with the insulin receptor (IR) and insulin signaling defects in hepatic IRS-1 tyrosine phosphorylation and phosphatidylinositol 3-kinase (PI3k) activation that may contribute to development of insulin resistance and subsequent development of type 2 diabetes mellitus [27, 28]. 

The hypothesis that HCV core protein can modify the metabolic profile of HCV infected patients which leads to development of type II diabetes mellitus is also supported by Experimental data derived from transgenic mice infected with hepatitis C core protein that recently demonstrated that this protein induces insulin resistance directly, and tends to occur early in the course of infection, prior to development of steatosis or fibrosis [29]. Among humans with chronic hepatitis C (CHC) infection, it has been shown that insulin signaling in the liver is altered by defects in IRS-1 tyrosine phosphorylation and phosphatidyl inositol 3kinase activation, thus possibly contributing to insulin resistance [30]. It is also suggested that the proinflammatory cytokine, TNF-*α*, may mediate this process. TNF-*α* is upregulated in patients with chronic hepatitis C (CHC) and this cytokine has been shown to interrupt insulin signaling via reduced-tyrosine phosphorylation of IRS-1 and decreased ability of IRS-1 to associate with the insulin receptor. Data to support a role for TNF-*α* in the genesis of insulin resistance found in insulin resistant transgenic mice infected with hepatic C core protein. When treated with anti-TNF-*α*, insulin sensitivity significantly improves [31, 32]. More recent evidence suggests that the hepatitis C virus may further alter insulin signaling by upregulating expression of the protein suppressor of cytokine signaling 3, resulting in decreased activation of downstream components of insulin receptor signaling (IRS), and altered expression of sterol regulatory binding protein 1c, which is important in *de novo* lipogenesis [19, 33]. 

In contrast, this study is not consistent with the studies that indicated a lower prevalence of HCV infection in type II diabetic patients. Some of these controlled and uncontrolled study reports which were done in Tunisia by Naoufel et al., 2009, Brazil by Luce Marina et al., 2008, Turkey by Gulcan et al., 2008, Nigeria by Williams et al., 2006 and Greece by Sotiropoulos et al., 2001 indicated that there was no statistical significant difference between cases and controls with respect to HCV prevalence with *P* value of >0.05 [17, 34–37]. The reason for this low prevalence of HCV in diabetes which was seen in these studies might be associated with three different factors, the first reason might be the lack of study power due to smaller sample size, the second reason might be in association with HCV genotype difference. Until now, it is known that there are seven genotypes of HCV distributed in different parts of the world and these seven genotypes might not be equally associated with diabetes [38]. The third reason might be due to the circulation of HCV at low level in particular community.

In this study, multivariate analysis of logistic regression to determine the risk factor for HCV revealed that none of HCV risk factor variables we computed were significantly associated with HCV sero positivity. This might be due to the difficulty in identifying the risk factor for HCV infection which is paucity in establishing the proper mode of transmission to HCV. This finding is in agreement with the findings of Williams et al. [17] and Chen et al. [24]. But as compared to Sotiropoulos et al., 2001, this finding is consistent only with hospital admission and house hold contact risk factors which have no association on both studies. Moreover, Sotiropoulos et al. demonstrated that history of transfusion is associated with HCV sero positivity (*P* < 0.001) which is different from our study. The possible reason for this might be due to very minimum number of transfused participants encountered in this study [17, 24, 37]. 

## 5. Conclusion

In this study, it is indicated that there is an association between HCV infection and diabetes mellitus. As we did not use HCV RNA, we could not say that HCV infection is a risk for development of diabetes. We further recommend use of HCV RNA test for a study designed with prospective cohort to be conducted for better extrapolation of HCV being a risk factor for diabetes type II.

## Figures and Tables

**Table 1:** Socio demographic and other risk factor variables distribution among diabetic and non diabetic study subjects, Jimma University Specialized Hospital, 2010.

Risk	Diabetics (*N* = 304)	Nondiabetics (*N* = 300)	*P*	Crude OR		Adjusted OR	
	Frequency (%)	Frequency (%)		COR	95% CI	AOR	95% CI
Sex							
Male	188 (61.8)	170 (56.7)		1		1	
Female	116 (38.2)	130 (43.3)	0.196	0.8	(0.583, 1.117)	0.682	(0.42, 1.106)
Age							
0–40	129 (42.4)	273 (91.0)		1		1	
>40	175 (57.6)	27 (9.0)	0.000	13.7	(8.69, 21.6)	9.476	(5.655, 15.879)
Work							
Sedentary	139 (45.7)	75 (25.0)		1		1	
Nonsedentary	165 (54.3)	225 (75.0)	0.000	0.396	(0.280, 0.559)	0.261	(0.150, 0.451)
Ethnicity							
Oromo	189 (62.2)	174 (58.0)		1		1	
Amhara	52 (17.1)	54 (18.0)	0.284	0.80	(0.542, 1.197)	1.793	(0.918, 3.501)
Others	63 (20.7)	72 (24.0)	0.712	0.90	(0.546, 1.512)	1.511	(0.759, 3.007)
Education							
Illiterate and informal educ.	105 (34.5)	69 (23.0)		1		1	
1–8	122 (40.1)	90 (30.0)	0.222	0.71	(0.413, 1.228)	0.447	(0.199, 0.1002)
9–12	38 (12.5)	105 (35.0)	0.406	0.79	(0.471, 1.356)	0.332	(0.153, 0.721)*
>12	39 (12.8)	36 (12.0)	0.000	2.99	(1.667, 5.376)	1.540	(0.698, 3.394)
BMI							
<18.5	50 (16.4)	12 (4)		1		1	
18.5–24.9	171 (56.3)	261 (87)	0.942	0.960	(0.321, 2.867)	0.290	(0.079, 1.063)
25–29.9	59 (19.4)	21 (7)	0.000	6.105	(2.45, 15.25)	3.137	(1.059, 9.286)*
>29.9	24 (7.9)	6 (2)	0.499	1.424	(0.511, 3.964)	1.198	(0.354, 4.048)
HCV							
Negative	274 (90.1)	290 (96.7)		1		1	
Positive	30 (9.9)	10 (3.3)	0.002	3.17	(1.523, 6.618)	2.997	(1.08, 8.315)

*P < 0.05, COR: crude odds ratio, BMI: body mass index, CI: confidence interval.

**Table 2:** Frequency distribution of HCV risk factors between diabetic and non diabetic controls at Jimma University Specialized Hospital, July 2010.

	Diabetes (*N* = 304)			Non diabetes (*N* = 300)		
	HCV +ve Frequency (%)	HCV −ve Frequency (%)	*P* value	HCV +ve Frequency (%)	HCV –ve. Frequency (%)	*P* value
Ear piercing						
Yes	9 (2.96)	100 (32.89)	0.48	6 (2.0)	133 (44.33)	0.38
No	21 (6.9)	174 (57.2)		4 (1.3)	157 (52.33)	
Tattoo						
Yes	6 (1.97)	33 (10.85)	0.22	1	37 (12.3)	0.797
No	24 (7.89)	241 (79.276)		9 (3.0)	253 (84.3)	
Tooth extraction						
Yes	11 (3.62)	113 (37.17)	0.629	2 (0.66)	55 (18.3)	0.94
No	19 (6.25)	161 (52.96)		8 (2.6)	235 (78.3)	
Hospital admission						
Yes	16 (5.26)	175 (57.56)	0.26	0 (0)	6 (2)	NA
No	14 (4.6)	99 (32.56)		10 (3.3)	284 (94.6)	NA
Contact with jaundiced.						
Yes	4 (1.32)	20 (6.58)	0.252	0 (0)	6 (2)	NA
No	26 (8.55)	254 (83.55)		10 (3.3)	284 (94.6)	NA
Body piercing						
Yes	4 (1.32)	15 (4.93)	0.103	0	9 (3)	NA
No	26 (8.55)	259 (85.2)		10 (3.3)	281 (93.6)	NA
Blood transfusion						
Yes	0	2 (0.7)	NA	0	0	NA
No	30 (9.9)	272 (89.5)	NA	10 (3.3)	290	NA

NA: not applicable.

**Table 3:** Unpaired *t*-test values for the difference of means between diabetic and non diabetic controls with equal variance is assumed, Jimma University Specialized Hospital, July 2010.

Variable	Diabetic Mean ± standard deviation	Non diabetic Mean ± standard deviation	Mean difference diabetic/non diabetes	*P* value	95% CI
Age in years	44.3 ± 15.3	26.87 ± 8.3	17.456	0.000	(15.49, 19.42)
Height (m)	1.68 ± 0.52	1.64 ± 0.099	0.0398	0.199	(−0.02, 0.1)
Weight (kg)	62.2 ± 13.5	59.5 ± 7.4	2.62	0.003	(0.88, 4.38)
BMI (kg/m^2^)	22.7 ± 4.79	22.08 ± 2.06	0.69	0.022	(0.098, 1.28)
AST (U/L)	33.46 ± 12.04	31.89 ± 4.76	1.56	0.037	(0.092, 3.03)
ALT (U/L)	34.76 ± 17.4	34.24 ± 10.5	0.52	0.656	(−1.783, 2.83)
Cholesterol (mg/dL)	197.05 ± 4.8	131.7 ± 2.06	65.33	0.000	(49.33, 81.34)

AST: aspartate transaminase, ALT: alanine transaminase.
